# Does the timing of parental migration matter for child growth? A life course study on left-behind children in rural China

**DOI:** 10.1186/s12889-015-2296-y

**Published:** 2015-09-25

**Authors:** Nan Zhang, Laia Bécares, Tarani Chandola

**Affiliations:** Room 3.331, The School of Nursing, Midwifery and Social Work, Jean McFarlane Building, The University of Manchester, Oxford Road, Manchester, M13 9 PL UK; Cathie Marsh Institute for Social Research (CMIST), School of Social Sciences, The University of Manchester, Oxford Road, Manchester, M13 9PL UK

**Keywords:** Internal migration, Left-behind children, Nutritional status, China, Life course

## Abstract

**Background:**

China’s unprecedented internal migration has left 61 million rural children living apart from parents. This study investigates how being left behind is associated with children’s growth, by examining children’s height and weight trajectories by age, testing the accumulation and critical period life course hypotheses.

**Methods:**

Data were drawn from five waves of the China Health and Nutrition Survey (CHNS). Multiple cohorts of children under 6 years old from 1997–2009 were examined (*N* = 2,555). Growth curve models investigated whether height and weight trajectories differ for children who were left behind at different stages of the life course: in early childhood (from ages 0–5 but not afterwards), in later childhood (from ages 6 to 17 only), and in both early and later childhood (from ages 0–5 and from ages 6–17), compared to their peers from intact households.

**Results:**

Boys who were left behind at different life stages of childhood differed in height and weight growth compared with boys from intact families. No significant associations were found for girls. As young boys turned into adolescents, those left behind in early childhood tended to have slower height growth and weight gain than their peers from intact households. There was a 2.8 cm difference in the predicted heights of boys who were left behind in early childhood compared to boys from intact households, by the age of 14. Similarly, the difference in weight between the two groups of boys was 5.3 kg by the age of 14.

**Conclusions:**

Being left behind during early childhood, as compared to not being left behind, could lead to slower growth rates of height and weight for boys. The life course approach adopted in this study suggests that early childhood is a critical period of children’s growth in later life, especially for boys who are left behind. The gender paradox in China, where sons are preferred, but being left behind appears to affect boys more than girls, needs further exploration.

## Background

@China’s unprecedented internal migration has left around 61 million rural children under 18 years of age living apart from either one or both parents, which accounts for 37.7 % of total rural children, and for 21.9 % of all children throughout China [1]. A growing number of cross-sectional studies suggest that left-behind children (LBC) in rural China are more likely to undergo restricted growth (i.e., stunting and underweight) than non-left-behind children (non-LBC) from intact families [2–6]. However, one survey in rural Southern China indicates that there are no differences in height between LBC and non-LBC aged 10–18, although LBC tend to be more likely to be overweight and/or obese than their peers [7]. Although they provide interesting findings, these studies are based on cross-sectional data and focus on relatively narrow age ranges, failing to capture the effects of parental migration on children’s nutritional health as they grow. They also confound age and cohort effects, and are prone to selection bias due to lack of adequate control for individual-level heterogeneity [8].

The limited longitudinal studies on LBC and growth report conflicting associations between parental migration and children’s nutritional health. A longitudinal analysis of the China Health and Nutrition Survey (CHNS) indicates that parental migration does not appear to significantly affect children’s overweight status, while older children (aged 7–12) of migrant parents are more likely to be underweight, as compared to non-left-behind peers [9]. In a later study examining the same dataset, Mu and de Brauw [10] investigate the impact of parental migration on nutritional status of children aged under 5 at baseline who were followed up in terms of height-for-age Z-scores (HAZ) and weight-for-age Z-scores (WAZ) and show that parental migration does not significantly affect young children’s HAZ, but it improves their WAZ. Although these studies improve on the cross-sectional limitations of early studies, both use fixed effects models that only analyse within-child change in height and weight, rather than between-child differences, failing to detect heterogeneity in growth between children according to their left behind status. This is an important limitation, as differences between LBC and children from intact households may increase as they get older.

Another limitation of existing longitudinal studies is the lack of consideration given to life course effects, as they fail to distinguish between the effects of parental migration at a critical period of the children’s life course (for example, during pre-school years), and do not investigate whether the effects of parental migration accumulate over the children’s life course, if they are left behind during separate spells as they grow up [11]. It may be hypothesised that being left behind at a younger age (for example, when under 6 years of age before the children start school) may have a stronger adverse effect on children’s subsequent growth than being left behind at later stages of childhood — a so-called ‘critical period hypothesis’ [12]. On the other hand, the effects of being left behind on children’s nutritional health may accumulate throughout the child’s life course, as argued by the accumulation hypothesis [13]. According to the accumulation hypothesis, being left behind by migrant parents in early and later childhood can have a cumulative effect on children’s growth and development, and can therefore be more detrimental than being left behind during a critical period.

The aim of the present study is to address the previous limitations in the literature and to investigate the associations between being left behind at different stages of childhood and children’s nutritional status in terms of physical development in relation to the accumulation and critical period hypotheses in life course epidemiology.

## Methods

### Data

The China Health and Nutrition Survey (CHNS) is an ongoing open cohort, international collaborative project between the Carolina Population Centre at the University of North Carolina at Chapel Hill and the National Institute of Nutrition and Food Safety at the Chinese Centre for Disease Control and Prevention. It employs a multistage, random-clustered sampling process to draw a sample of about 4,400 households with a total of about 19,000 participants from over 200 communities or neighbourhoods in nine provinces, with the first round conducted in 1989. Although the CHNS is not a nationally representative sample [14], the counties within selected provinces were chosen to represent a range of income level, and provinces vary substantially in geography, economic development, public resources, and health indicators.

The survey has been approved by the institutional review committees from the University of North Carolina at Chapel Hill and the National Institute for Nutrition and Food Safety, China Centre for Disease Control and Prevention. All participants and/or their parents/guardians provided written informed consent for their participation in the survey.

### Target population

This study used data from the five waves of the CHNS, collected in 1997, 2000, 2004, 2006, and 2009. The status of being left behind was operationalised using the household roster: from 1997 onwards, if one household member in a previous round of the CHNS was not residing in the same household in the current survey, the respondent was asked for the reasons for his/her absence. In this study, children under 18 years old whose parent (s) have left the home to seek employment elsewhere are defined as LBC. Children varying in age (between 0 and 6) were recruited in 1997 and then followed up for 12 years up to 2009, by drawing on accelerated longitudinal designs [15]. This allows us to explore age-outcome trajectories over a broader age span (between 0 and 18) during a relatively shorter study period (from 1997–2009). In addition, we also followed up multiple cohorts of the age range between 0 and 6 in 2000, 2004, 2006 and 2009. Multiple cohorts at different waves included not only new-born eligible children, but also a new province that was added from 2000, and villages lost to follow-up returned in later waves [14]. Children with non-missing values on outcome variables and key predictors were kept in the analysis, yielding a total sample of 1,231 children with 2,555 observations.

### Variables

Child growth is an important public health indicator for monitoring nutritional status and health amongst children [16]. Poor nutritional health, regardless of its aetiological origins, can affect growth to some extent [17]. Anthropometric measurement (e.g., height and weight) is highly recommended to assess nutritional status for children and adolescents [18]. The CHNS recorded height and weight for each individual within the household, measured by health professionals. Height and weight were used as outcome variables.

To examine critical period and cumulative effects, we created a four-category time-invariant variable to indicate at which stage of childhood years the child was left behind, ranging from ages 0–17: children from intact households who were never left behind and were the reference group; children who were left behind only in early childhood, from ages 0–5, but not in later childhood during school years, from ages 6–17; children who were left behind in both early and later childhood; and children who were left behind only in later childhood, from ages 6–17, but not from ages 0–5.

We adjusted for a range of demographic and socioeconomic factors, including age, gender, insurance status (whether a child has insurance or not), only child (whether a child is the only child within a household), household size, annual household income per capita, maternal education (the number of years of formal education completed), and maternal height and weight. We also included wave dummies and province dummies to capture time and geographical effects respectively.

Given the possible recall bias in income data from CHNS, we created a household asset score using principal component analysis on the household items that mainly included having a colour television, washing machine, air conditioner, tap water, and flush toilet. This asset score was used as a measure of socio-economic status for each household at each time point from 1997–2009. Compared with household income or expenditure data, asset-based measures of socio-economic status are more reflective of longer-term household wealth or living standards [19, 20].

### Analysis

Growth curve models, which model individual differences in change/growth over time, are appropriate to use with data where repeated measurement occasions are clustered within participants [8] because they take into account the dependence of residuals due to covariance between the levels in the data. Failure to account for the inter-dependence of residuals can underestimate standard errors, leading to biased estimates. A key advantage of growth curve models is that they can be estimated in the presence of unbalanced and incomplete data under a Missing At Random (MAR) assumption (missingness is not associated with the value of missing variable itself but associated with other observed variables) [21]. All children whose height or weight was measured on at least one occasion were included in the analysis. Only missing occasions were automatically removed from growth curve models, rather than children with any missing data.

The growth curve models can be expressed by equations at two levels and fitted by using MLwiN Version 2.28 [22], within multiple occasions (Level 1) nested within children (Level 2) over time. We also run three-level models further accounting for household level (Level 3) as there may be more than one child within the same household. And the household-level clustering effects were found to be relatively small and statistically insignificant. Hence we reported two-level growth curve models in this paper and assumed that the household clustering effects are negligible. Children’s age was used as the indicator of time metric. In order to facilitate parameter interpretation, we centred age at the grand mean.

The individual growth model or level (1) submodel takes the following form:

1 $$ {H}_{ij}={\pi}_{0j}+{\pi}_{1j}Ag{e}_{ij}+{\pi}_{2j}Ag{e}_{ij}^2+{\pi}_3{D}_{ij}+{\varepsilon}_{ij} $$

Where *H*_*ij*_ is the nutritional status in terms of height and weight measured for the *j*th (*j* = 1,2,...,N) child at occasion *i* (*i* = 1, 2,...,T). Use of a quadratic function, *Age*_*ij*_ and *Age*_*ij*_^2^ allows for non-linear changes so that the effects of age on *H*_*ij*_ can increase or decrease over time. *π*_0*j*_ denotes *j*th child’s nutritional status at mean age. *π*_1*j*_ captures linear growth rate and *π*_2*j*_ captures the curvilinearity of the growth trajectory and are allowed to vary between children, so that the model estimates different growth curves for each child. *D*_*ij*_ represents a set of time-varying covariates; its effects on children’s nutritional status are denoted as *π*_3_. *ε*_*ij*_ is the Level-1 residuals.

We would expect that the children’s nutritional status at mean age is likely to be confounded by background time-invariant predictors that bear on children’s nutritional status, such as only child, and being left behind at different stages of childhood.

The level (2) submodel can be written as:

2.1 $$ {\uppi}_{0j}={\uppi}_{00}+{\uppi}_{01}{\mathrm{X}}_j+{\uppi}_{02}{\mathrm{M}}_j+{U}_{0j} $$

where π_00_ indicates the average nutritional status at mean age. *X*_*j*_ represents other time-invariant covariates at the individual level, other than being left behind at different stages, and ${\uppi}_{01}$ represents its effects on children’s nutritional status at the mean age. M_j_ is a four-category time-invariant variable denoting the stage of childhood when the child was left behind: the reference group is children from intact families who were never left behind; and another three categories refer to children who were left behind in early childhood only, children who were left behind in both early and later childhood, and children who were left behind in later childhood only respectively. π_02_ represents its effects on children’s nutritional status at mean age. *U*_0*j*_ represents how the *j*th child’s nutritional status at mean age deviates from the average initial level π_00_.

To examine whether growth rates of nutritional status differ between children left behind at different stages of childhood compared to children from intact families, we estimate the interactions between the left behind stage variables and children’s age (linear and quadratic terms).

The Level-2 submodels for growth rates take the following forms:

2.2 $$ {\uppi}_{1j}={\uppi}_{10}+{\uppi}_{12}{\mathrm{M}}_j+{U}_{1j} $$

2.3 $$ {\uppi}_{2j}={\uppi}_{20}+{\uppi}_{22}{\mathrm{M}}_j+{U}_{2j} $$

Where π_12_ and π_22_ indicate non-linear associations between being left behind at different stages of childhood and growth rates of children’s nutritional status. *U*_1*j*_ and *U*_1*j*_ are individual-specific random effects: *U*_1*j*_ and *U*_2*j*_ indicating how the linear growth rate and the quadratic growth rate vary in accordance with the average linear growth term *π*_10_ and the average quadratic growth rate π_20_ respectively at the mean age.

The model assumptions are expressed as:

3.1 $$ {\upvarepsilon}_{\mathrm{ij}}\sim \mathrm{N}\left(0,{\upsigma}^2\right) $$

3.2 $$ \left(\begin{array}{l}{U}_{0j}\\ {}{U}_{1j}\\ {}{U}_{2j}\end{array}\right)\sim N\left\{\begin{array}{c}\hfill \hfill \\ {}\hfill \hfill \\ {}\hfill \hfill \end{array}\left[\begin{array}{c}\hfill 0\hfill \\ {}\hfill 0\hfill \\ {}\hfill 0\hfill \end{array}\right],\left[\begin{array}{c}\hfill {\sigma}_0^2\hfill \\ {}\hfill {\sigma}_{10}\hfill \\ {}\hfill {\sigma}_{20}\hfill \end{array}\begin{array}{c}\hfill {\sigma}_{10}\hfill \\ {}\hfill {\sigma}_1^2\hfill \\ {}\hfill {\sigma}_{21}\hfill \end{array}\begin{array}{c}\hfill {\sigma}_{20}\hfill \\ {}\hfill {\sigma}_{21}\hfill \\ {}\hfill {\sigma}_2^2\hfill \end{array}\right]\begin{array}{c}\hfill \hfill \\ {}\hfill \hfill \\ {}\hfill \hfill \end{array}\right\} $$

Cov (ε_ij_, *U*_*oj*_) = 0, Cov (ε_ij_, *U*_1*j*_) = 0, Cov (ε_ij_, *U*_2*j*_) = 0

where Level-1 residuals ε_ij_ are assumed to follow a normal distribution, Level-2 residuals *U*_0*j*_, *U*_1*j*_, and *U*_2*j*_ are assumed to follow a multivariate normal distribution, and Level-1 residuals are independent of Level-2 residuals. Equation 3.2 presents variance-covariance matrix among the Level-2 residuals. *σ*_0_^2^, *σ*_1_^2^, and *σ*_2_^2^ denote variances for children’s nutritional status at mean age, the average linear growth rate, and the average quadratic growth rates, respectively. *σ*_10_ and *σ*_20_ indicate covariances for children’s nutritional status with linear and quadratic growth rates. *σ*_20_ represents covariance between linear and quadratic growth rates.

The better fit of the models is associated with lower values in −2 log likelihood statistics. Nested models are compared through deviance statistics (difference in −2 log likelihood) over the difference in degrees of freedom using an ordinary chi-square distribution [8]. A significant difference between two nested models indicates that the models with the lowest value have a better fit to the data. Given the sex difference in physical development [23], we perform subgroup analyses based on stratification by gender for boys and girls separately.

## Results

Table 1 describes the nutritional outcomes in terms of height and weight, and socio-economic characteristics of boys and girls aged 0–17 from 1997–2009. In general, boys were more likely than girls to be the only child within a household. Boys were more likely than girls to be left behind in early childhood except in 1997 and 2004. Children’s economic status in terms of household income per capita and asset score improved over time, and girls tended to be better off than boys. There were no significant gender differences for height, weight and age.

**Table 1:** Characteristics and health outcomes of children (aged 0–17) by gender in rural China

	Wave					
	1997	2000	2004	2006	2009	N
*Boys*	*Proportion (%)*					
Only child^a^	37 (22.0)	55 (22.5)	97 (28.5)	118 (34.7)	188 (50.7)	495
Never left behind	120 (71.4)	157 (64.1)	226 (66.3)	217 (63.8)	249 (67.1)	969
Left behind in early childhood only	4 (2.4)	16 (6.5)	35 (10.3)	47 (13.8)	67 (18.1)	169
Left behind in early and later childhood	5 (3.0)	14 (5.7)	22 (6.5)	19 (5.6)	18 (4.9)	78
Left behind in later childhood only	39 (23.2)	58 (23.7)	58 (17.0)	57 (16.8)	37 (10.0)	249
*Mean (s.d.)*						
Height (cm)	93.6 (13.7)	103.2 (17.5)	114.8 (24.6)	117.9 (27.0)	120.3 (30.5)	1465
Weight (kg)	14.3 (3.6)	17.4 (5.6)	22.6 (10.0)	24.4 (12.1)	26.179 (15.0)	1465
Age (years)	3.6 (1.7)	5.1 (2.5)	7.0 (3.7)	7.5 (4.2)	7.6 (4.8)	1465
Household income per capita (RMB)	3100.7 (2735.1)	3508.4 (2741.4)	4594.5 (4088.1)	4938.8 (5103.2)	8471.8 (7968.2)	1465
Asset score	1.2 (0.7)	1.2 (0.8)	1.6 (0.8)	1.7 (0.8)	1.8 (0.8)	1465
*Girls*	*Proportion (%)*					
Only child^a^	26 (23.4)	32 (18.4)	61 (23.6)	85 (30.9)	102 (37.5)	306
Never left behind	82 (73.9)	112 (64.4)	166 (64.3)	179 (65.1)	182 (66.9)	721
Left behind in early childhood only	7 (6.3)	8 (4.6)	27 (10.5)	37 (13.5)	38 (14.0)	117
Left behind in early and later childhood	4 (3.6)	10 (5.8)	17 (6.6)	16 (5.8)	16 (5.9)	63
Left behind in later childhood only	18 (16.2)	44 (25.3)	48 (18.6)	43 (15.6)	36 (13.2)	189
	*Mean (s.d.)*					
Height (cm)	90.5 (14.9)	103.0 (17.2)	112.5 (25.3)	116.8 (26.9)	120.8 (27.2)	1090
Weight (kg)	13.2 (3.7)	16.8 (5.1)	21.4 (9.8)	24.0 (12.4)	25.9 (13.7)	1090
Age (years)	3.3 (1.8)	5.2 (2.4)	6.7 (3.7)	7.4 (4.2)	8.0 (4.7)	1090
Household income per capita (RMB)	3232.9 (2796.2)	3593.2 (3626.2)	4600.4 (4162.2)	5416.7 (5865.4)	7158.6 (6862.7)	1090
Asset score	1.3 (0.7)	1.2 (0.7)	1.6 (0.8)	1.7 (0.8)	1.7 (0.8)	1090

^a^Whether this child is the only child within a household

**Fig. 1:**
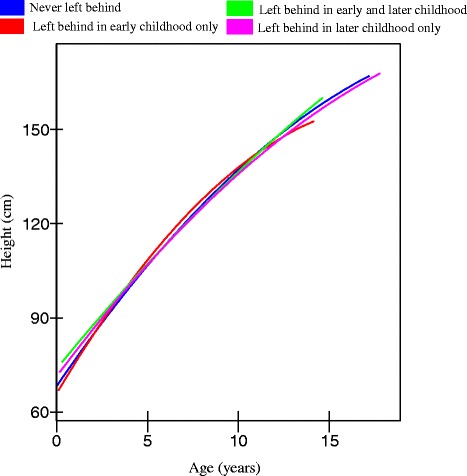
Trajectories of height for boys in rural China (estimated from model 2, Table 2)

Table 2 presents the coefficients and standard errors of the quadratic growth curve models (with age and age^2^ terms) of height separately for boys and girls. Among boys, Model 1 shows that boys from intact families were 115.7 cm tall on average at the age of 7, and grew about 6.4 cm (6.58–0.14) from age 7 to 8. Being left behind at different stages of childhood was significantly associated with height (*p* < 0.01) and the interactions with the linear and quadratic terms denoting children’s age were also significant (*p* = 0.01). Furthermore, the interactions between being left behind and the age terms remained significant even after adjusting for a range of socio-economic and socio-demographic confounders (*p* < 0.01) (Model 2). The predicted heights for boys at different ages from Model 2, Table 2 are shown in Fig. 1. On average, by teenage years, boys who were left behind before the age 6 appeared to grow not as tall as boys who were never left behind. By the age of 14, boys who were never left behind were 153.7 cm tall, on average, while boys who were left behind before the age of 6 were only 150.9 cm tall. Another measure of the difference between the two groups of boys was the average age by which they reached a height of 150 cm — 13.1 years for boys who were never left behind and 13.7 years for boys who were left behind in early childhood. Boys who were left behind in later childhood (after the age of 6) also were not as tall as boys who were never left behind by the age of 14, although this difference was not as large as the difference between those left behind at an early age and those never left behind. Boys who were left behind in both early and later childhood periods had the most favourable height growth trajectory. But this group was small (*N* = 78), thus our estimate may be unreliable.

**Table 2:** Estimates (standard errors) of height and growth curve models fitted to boys and girls

	Boys		Girls	
Fixed effects	Model 1^a^	Model 2^b^	Model 1^a^	Model 2^b^
Intercept	115.70 (0.35)	116.68 (0.96)	115.76 (0.41)	117.14 (1.05)
Age (mean centred at age 7)	6.58 (0.06)	6.41 (0.07)	6.67 (0.06)	6.55 (0.07)
Age^2^	−0.14 (0.01)	−0.16 (0.01)	−0.19 (0.01)	−0.20 (0.01)
Left-behind stage (reference group never left behind child aged 7)				
Never left behind	0	0	0	0
In early childhood only	2.62 (0.91)	1.99 (0.80)	−0.82 (1.09)	−0.21 (0.93)
In early and later childhood	−1.51 (1.25)	−0.25 (1.05)	−1.94 (1.42)	0.06 (1.17)
In later childhood only	−1.64 (0.79)	−0.40 (0.66)	−2.03 (0.92)	−0.40 (0.76)
*Deviance statistics*	16.63	7.15	6.10	0.31
*P-value for Chi-square (df = 3)*	<0.01	0.07	0.11	0.96
Interactions (reference group never left behind child aged 7):				
Age × Left-behind stage:				
In early childhood only	0.17 (0.23)	0.01 (0.22)	−0.42 (0.21)	−0.46 (0.20)
In early and later childhood	−0.23 (0.23)	−0.38 (0.23)	0.02 (0.22)	−0.19 (0.23)
In later childhood only	−0.35 (0.14)	−0.36 (0.14)	0.03 (0.15)	−0.08 (0.16)
Age^2^ × Left-behind stage:				
In early childhood only	−0.09 (0.04)	−0.10 (0.04)	0.01 (0.04)	0.00 (0.04)
In early and later childhood	0.07 (0.05)	0.08 (0.05)	0.05 (0.05)	0.05 (0.05)
In later childhood only	0.03 (0.03)	0.03 (0.03)	−0.00 (0.03)	−0.00 (0.03)
*Deviance statistics*	18.31	18.94	5.86	6.79
*P-value for Chi-square (df = 6)*	0.01	<0.01	0.44	0.34
−2 Log likelihood	9357.00	9138.48	6872.58	6681.72

^a^Adjusts for age, age^2^, left-behind stages, and the interaction effects between age × left-behind stage, and age^2^ × left-behind stage

^b^Additionally adjusts for covariates, e.g., gender, only child, child insurance, maternal education, maternal height, household size, household income per capita, asset score, wave, and province, which are not reported here

**Fig. 2:**
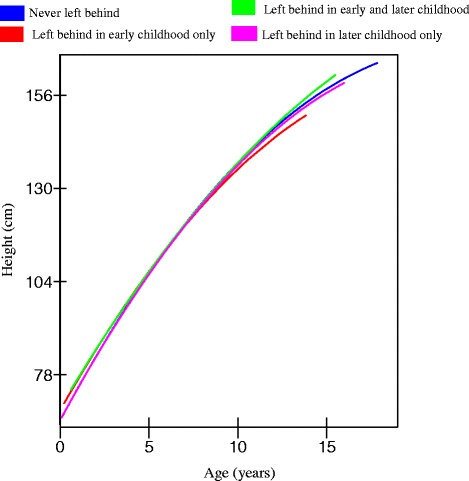
Trajectories of height for girls in rural China (estimated from model 2, Table 2)

For girls, being left behind was not significantly associated with height, nor were they any significant interactions with age and age square (Table 2). The predicted heights for girls (Fig. 2) suggested girls who were left behind before the age of 6 were shorter, on average, than never left behind girls, by teenage years, although these differences were not significant.

**Fig. 3:**
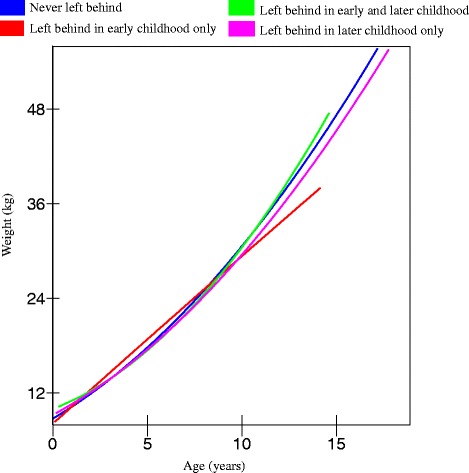
Trajectories of weight for boys in rural China (estimated from model 2, Table 3)

**Fig. 4:**
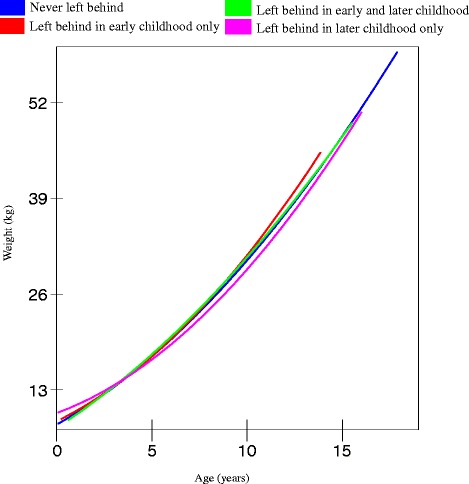
Trajectories of weight for girls in rural China (estimated from model 2, Table 3)

A similar gender difference emerged for the trajectories of weight gain. Being left behind at different stages of childhood was significantly associated with lower weight gain among boys (*p* = 0.03 in Model 2, Table 3), but not among girls. Once again, boys who were left behind before the age of 6 had the slowest increase in weight by the age of 14 (36.7 kg) compared to boys who were never left behind (42 kg), as shown in Figs. 3 and 4. Boys who were never left behind had a weight of 36 kg, by the age of 12.2 years, on average, while boys who were left behind in early childhood reached that weight when they turned 13.7 years old, on average. Boys who were left behind in later childhood also had slower weight gain trajectories than boys who were never left behind. Boys who were left behind in both early and later childhood periods had the most favourable weight increase trajectory. But as this was a small group (*N* = 78), our estimate may be unreliable.

**Table 3:** Estimates (standard errors) of weight and growth curve models fitted to boys and girls

	Boys		Girls	
Fixed effects	Model 1^a^	Model 2^b^	Model 1^a^	Model 2^b^
Intercept	20.91 (0.18)	21.20 (0.41)	20.76 (0.24)	21.24 (0.50)
Age (mean centred at age 7)	2.46 (0.05)	2.41 (0.05)	2.57 (0.06)	2.48 (0.06)
Age^2^	0.08 (0.01)	0.08 (0.01)	0.09 (0.01)	0.08 (0.01)
Left-behind stage (reference group never left behind child aged 7)				
Never left behind	0	0	0	0
In early childhood only	0.71 (0.50)	0.76 (0.47)	0.06 (0.65)	0.10 (0.58)
In early and later childhood	−1.04 (0.65)	−0.49 (0.59)	−0.15 (0.84)	0.40 (0.74)
In later childhood only	−0.89 (0.41)	−0.49 (0.37)	−1.28 (0.55)	−0.81 (0.48)
*Deviance statistics*	10.26	5.72	5.66	3.51
*P-value for Chi-square (df = 3)*	0.02	0.13	0.13	0.32
Interactions (reference group never left behind child aged 7):				
Age × Left-behind stage:				
In early childhood only	−0.30 (0.20)	−0.30 (0.20)	0.13 (0.19)	0.10 (0.19)
In early and later childhood	−0.01 (0.16)	−0.03 (0.16)	0.08 (0.19)	0.05 (0.18)
In later childhood only	−0.21 (0.10)	−0.16 (0.10)	−0.20 (0.11)	−0.21 (0.11)
Age^2^ × Left-behind stage:				
In early childhood only	−0.08 (0.03)	−0.08 (0.03)	0.03 (0.03)	0.02 (0.03)
In early and later childhood	0.04 (0.03)	0.04 (0.03)	−0.02 (0.03)	−0.01 (0.03)
In later childhood only	−0.00 (0.02)	−0.00 (0.02)	0.01 (0.02)	0.02 (0.02)
*Deviance statistics*	15.97	13.89	6.42	7.15
*P-value for Chi-square (df = 6)*	0.01	0.03	0.38	0.31
−2 Log likelihood	7567.18	7416.62	5709.49	5589.92

^a^Adjusts for age, age^2^, left-behind stages, and the interaction effects between age × left-behind stage, and age^2^ × left-behind stage

^b^Additionally adjusts for covariates, e.g., gender, only child, child insurance, maternal education, maternal weight, household size, household income per capita, asset score, wave, and province, which are not reported here

## Discussion

The present study aimed to examine the importance of being left-behind on boys’ and girls’ height and weight growth, and to explore the accumulation and critical period hypotheses to the exposure of parental migration in children’s nutritional status in terms of physical development. We found that boys from intact households tended to be better off in terms of height and weight growth compared to boys who were left behind only during early childhood, or only during later childhood, thus finding some support for the critical period hypothesis. There was a 2.8 cm difference in the predicted heights of boys who were left behind in early childhood compared to boys from intact households, by the age of 14. Similarly, the difference in weight between the two groups of boys was 5.3 kg by the age of 14. The accumulation hypothesis was not supported since the adverse effects of being left behind did not increase as the children aged. However, this finding should be treated with caution as the group of boys and girls who were left behind both in early and later childhood was very small (*N* = 78 for boys and *N* = 63 for girls).

Previous longitudinal evidence by drawing on CHNS data shows that parental migration does not affect young children’s HAZ (aged under 5 years old at baseline and followed up once up to age 9), but it improves their WAZ [10], and that LBC aged 7–12 are more likely to be underweight compared to their non-left-behind peers [9]. Our study suggests that being left behind in early childhood (from birth to 5 years old) can negatively affect boys’ height and weight growth by adolescence. The discrepancy between our results and others’ can partially derive from different age ranges of children, and nutritional outcomes. For example, our study targeted at children of a relatively wider age range, from birth to 18 years old, compared to other studies. We used height and weight as nutritional outcomes, rather than adjusted height and weight by age and gender, such as HAZ and WAZ as they are less straightforward to interpret [24]. More importantly, previous studies [9, 10] by using fixed effects models, tended to focus on within-child change of height and weight over relatively shorter time periods, but to neglect between-child differences regarding to their left behind status. These differences between LBC and children from intact households may increase with age with much larger gaps observed by a later stage of childhood (for example, by teenage years). The use of the life course approach in our study allows us to detect that being left behind in early childhood can lead to slower height growth and weight gain for boys by teenage years.

To our knowledge, the present study is the first attempt to employ a life course approach to explore the long-term associations between being left behind due to paternal migration and children’s nutritional status. A number of studies have consistently indicated that growth restriction in the early years of life often leads to several poor outcomes in adulthood [25–29]. Accordingly, life course studies suggest that health inequalities in adulthood, to some extent, begin in the early years of life [30, 31]. These two aspects of the literature provide some insights into how being left behind can shape health across the life course. We identified that the pre-school ages, from birth to 5 years, constitute critical periods for the exposure to parental migration in height growth and weight gain for boys. That is, an early life health shock in terms of parental migration can have long-term adverse effects on children’s nutritional outcomes.

Although no direct comparisons were made, we were still able to observe gender differences in the effects of being left behind at different stages on height and weight outcomes. For girls, being left behind at any time during childhood did not appear to affect their growth trajectories, while boys who were left behind in the preschool years had the slowest growth trajectories. This suggests that being left behind appears to be more detrimental for boys than girls. This may be partly attributed to the natural selection of physiological traits that increases female survival, which may render greater vulnerability of males to health insults such as parental migration in early life [32]. Some evidence, however, suggest the effect of parental migration can be more detrimental for girls than for boys in the Chinese context. Parental migration results in increased physical workload [33, 34] and time use on farm work and domestic work for left-behind girls aged 7–14, but not for boys of the same age range [35]. Moreover, girls are more likely than boys to be disadvantaged in nutrient intakes due to China’s ‘son preference’ norm, especially in rural areas [36]. Son preference in rural China contributes to additional height growth advantage for boys, and this effect becomes more pronounced in the teenage years [37]. Our study does not directly compare the effect of parental migration among boys and girls. Thus, there seems to a gender paradox in China, where sons are preferred, but being left behind appears to affect them more than girls. This needs further exploration.

The pathways through which migration affects children’s growth can be complex and multi-factorial. For example, remittances from migrant parents can enhance LBC’s economic status to improve access to healthcare services, healthy living environments, and nutritious food. However, one negative aspect of parental migration is family dissolution, which may expose LBC to adverse effects of psychological and emotional wellbeing [38]. These psychological symptoms may potentially affect dietary attitudes and cause unhealthy behavioural problems like smoking in adolescence, which could jeopardize LBC’s nutritional status [39–43]. Unfortunately, we were not able to distinguish the different mechanisms through which migration affects children’s nutritional status. Regardless of possible pathways, it can be useful and important to be able to identify and narrow down the specific ages at which the window of opportunity opens in order to facilitate interventions. Identifying the specific ages of critical period in childhood is a first step to design and deliver adequate interventions.

The limitations of this study mainly relate to data issues given that the CHNS was not originally designed to study internal migration and LBC. First, the study period we looked at is from 1997–2009, making use of five waves (1997, 2000, 2004, 2006 and 2009). We identified LBC according to the migration status of parents based on particular time points. However, parental migration status could have changed between waves, for example, old parental return or new parental migration between two time points, which were not tracked by the CHNS. This could lead to an underestimation of the LBC sample. Furthermore, we might have underestimated the samples of children who were left behind only in later childhood (aged 6–17) and those left behind throughout childhood (aged 0–5 and aged 6–17). We focused on multiple cohorts (aged 0–6) at each wave but were unable to follow all of them up to age 18 due to the limited time span (from 1997–2009). Second, previous studies suggest that being left behind by different parents may have different impacts on children’s nutritional status [7, 44, 45]. The present study did not distinguish whether children are left behind by the father, the mother, or by both parents because of the small numbers in these categories. Third, even though we tried to adjust for as many relevant confounders as possible, there are still certain important time-varying factors that were not captured by the CHNS, including remittances from migrant parent (s) [46] as well as the caregiving arrangements for children left behind [47–49]. This is particularly relevant for the children who were left behind at an early age — the families of such children may be much more disadvantaged than other groups of children and we may not have been able to adequately control for such disadvantage in our analyses.

And finally, one main limitation with this, and any longitudinal dataset, is missing data and sample attrition. In the CHNS, older children may not take part in later surveys, and school children who were in boarding schools, and who subsequently entered colleges and universities, may miss certain rounds of survey. Also, children may themselves migrate when aged above 16 years old [14]. Sample attrition and missing data could thus lead to biased results. We examined predictors of sample attrition and missingness in our study and found several variables that were related to attrition/missingness (results not shown), such as household income per capita, household size, child age, whether a child is the single child within his/her household, and asset score. These predictors could cause either downward or upward bias in our estimates. For example, it suggested that poorer children were more likely to drop out from subsequent surveys as they might have experienced more difficulties to participate. Given poorer children may have slower growth rates than richer children, so the attrition of poorer children can lead to a downward bias in our estimate of the effect of being left behind on children’s growth rates. We also found that children from larger families were less likely to drop out and a larger family size could be more detrimental to children’s growth rates due to restricted resource allocation, so our estimates could have been overestimated. Therefore, we were unable to distinguish the exact direction of bias that was introduced by sample attrition and missingness in our estimates. This is a major limitation of this study. An accelerated longitudinal design enabled us to track age-outcome trajectories over the entire childhood (from ages 0 to 17) during a relatively shorter study period (from 1997–2009). However, one danger of an accelerated longitudinal design is that it assumes there are no age-by-cohort interaction effects; or in other words, it assumes that a single growth trajectory can represent all the cohorts [15]. In fact, such effects may arise due to demographic differences (i.e., age, family background) between cohorts, and perhaps due to effects of history [50]. We adjusted for time effects by adding multiple baseline waves to minimise cohort differences associated with historical time in the age-outcome relationship but data pooled from multiple cohorts ignoring demographic differences may lead to biased inferences.

## Conclusion

Being left behind due to parental migration during early childhood, as compared to not being left behind, could lead to slower height growth and weight gain for boys. In contrast to previous findings that suggest contradicting effects of being left behind on children’s growth, the life course approach adopted in this study suggests that early childhood is a critical period for children’s growth in later life, especially for boys who are left behind. The gender paradox in China, where sons are preferred, but being left behind appears to affect boys more than girls, needs further exploration.

## Abbreviations

LBC: Left-behind children
Non-LBC: Non-left-behind children
CHNS: China health and nutrition survey
HAZ: Height-for-age Z-scores
WAZ: Weight-for-age Z-scores

## References

[1] All-China Women’s Federation: Research report on left-behind children and migrant children in China [http://acwf.people.com.cn/n/2013/0510/c99013-21437965.html]

[2] Chen C, He W, Wang Y, Deng L, Jia F (2011). Nutritional status of children during and post-global economic crisis in china. Biomed Environ Sci.

[3] Wei S, Ju L, Li M, Wang W (2011). Child health and nutrition: getting better and facing new challenges in China. Australasian Medical Journal.

[4] Duan D, Zhu M, Luo J, Wang Z, Gu C, Zhang W (2009). Investigation on dietary nutrients among rural stranded children of 2–7 year olds in China. Zhonghua Liu Xing Bing Xue Za Zhi.

[5] Mou J, Luo J, Li Y, Shuai Z, Liu X (2009). Study on the nutritional status and determinants among rural stranded children in China. Zhonghua Liu Xing Bing Xue Za Zhi.

[6] Luo J, Peng X, Zong R, Yao K, Hu R, Du Q (2008). The status of care and nutrition of 774 left-behind children in rural areas in China. Public Health Rep.

[7] Gao Y, Li LP, Kim JH, Congdon N, Lau J, Griffiths S (2010). The impact of parental migration on health status and health behaviours among left behind adolescent school children in China. BMC Public Health.

[8] Singer JD, Willett JB: Applied longitudinal data analysis: Modeling change and event occurrence: Oxford university press; 2003

[9] De Brauw A, Mu R (2011). Migration and the overweight and underweight status of children in rural China. Food Policy.

[10] Mu R, De Brauw A: Migration and young child nutrition: evidence from rural China. Journal of Population Economics. 2015;28(3):631-657.

[11] Spallek J, Zeeb H, Razum O: What do we have to know from migrants’ past exposures to understand their health status? a life course approach. *Emerg Themes Epidemiol* 2011, 8(6). 10.1186/1742-7622-8-6 PMC3169503 21843354

[12] Barker DJP (1998). Mothers, babies and health in later life, 2nd edn: Churchill Livingstone.

[13] Kuh D, Ben-Shlomo Y, Lynch J, Hallqvist J, Power C (2003). Life course epidemiology. J Epidemiol Community Health.

[14] Popkin BM, Du S, Zhai F, Zhang B (2010). Cohort Profile: The China Health and Nutrition Survey—monitoring and understanding socio-economic and health change in China, 1989–2011. Int J Epidemiol.

[15] Collins LM (2006). Analysis of longitudinal data: The integration of theoretical model, temporal design, and statistical model. Annu Rev Psychol.

[16] De Onis M, Blössner M (2003). The World Health Organization global database on child growth and malnutrition: methodology and applications. Int J Epidemiol.

[17] De Onis M, Habicht J-P (1996). Anthropometric reference data for international use: recommendations from a World Health Organization Expert Committee. Am J Clin Nutr.

[18] World Health Organization (1995). Physical status: the Use and interpretatioin of anthropometry.

[19] Filmer D, Pritchett LH (2001). Estimating wealth effects without expenditure Data—Or tears: An application to educational enrollments in states of india. Demography.

[20] Vyas S, Kumaranayake L (2006). Constructing socio-economic status indices: how to use principal components analysis. Health Policy Plan.

[21] Curran PJ, Obeidat K, Losardo D (2010). Twelve frequently asked questions about growth curve modeling. J. Cogn. Dev..

[22] Rasbash J, Charlton C, Browne WJ, Dealy M, Cameron B: MLwiN Version 2.28. In. Edited by Centre for Multilevel Modelling University of Bristol; 2013.

[23] Sinclair DC, Dangerfield P (1998). Human growth after birth, 6th edn: Oxford Univ Press.

[24] Wang Y, Chen H-J: Use of percentiles and Z-scores in anthropometry. In: *Handbook of Anthropometry: Physical Measures of Human Form in Health and Disease.* edn. Edited by Preedy V; 2012: 29–48.

[25] Grantham-McGregor S, Cheung YB, Cueto S, Glewwe P, Richter L, Strupp B (2007). Developmental potential in the first 5 years for children in developing countries. The Lancet.

[26] Mendez MA, Adair LS (1999). Severity and timing of stunting in the first two years of life affect performance on cognitive tests in late childhood. J Nutr.

[27] World Health Organization (1999). A critical link. Interventions for physical growth and psychological develoment: a review.

[28] Jukes M, McGuide J, Method F, Sternberg R: Nutrition and Eudcation. Nutrition: A Foundation for Development. In. Geneva, ACC/SCN; 2002.

[29] McCarthy M (1997). Stunted children are at high risk of later obesity. The Lancet.

[30] Braveman P, Barclay C (2009). Health disparities beginning in childhood: a life-course perspective. Pediatrics.

[31] Hallqvist J, Lynch J, Bartley M, Lang T, Blane D (2004). Can we disentangle life course processes of accumulation, critical period and social mobility? An analysis of disadvantaged socio-economic positions and myocardial infarction in the Stockholm Heart Epidemiology Program. Soc Sci Med.

[32] Wells JC (2000). Natural selection and sex differences in morbidity and mortality in early life. J Theor Biol.

[33] Ye J, Pan L (2011). Differentiated childhoods: impacts of rural labor migration on left-behind children in China. Journal of Peasant Studies.

[34] Ye J, Murray J, Wang Y (2005). Left-behind Children in Rural China: Impact Study of Rural Labor Migration on Left-behind Children in Mid-West China.

[35] Chang H, Dong X-y, MacPhail F (2011). Labor Migration and Time Use Patterns of the Left-Behind Children and Elderly in Rural China. World Dev.

[36] Ning M, Chang H-h: Migration decisions of parents and the nutrition intakes of children left at home in rural China. *Agricultural Economics/Zemedelska Ekonomika* 2013, 59(10):467-477.

[37] Song S, Burgard SA (2008). Does son preference influence children’s growth in height? A comparative study of Chinese and Filipino children. Popul Stud.

[38] Shi Y (2011). Three Essays on Economics of Health Behavior in China.

[39] Martyn-Nemeth P, Penckofer S, Gulanick M, Velsor-Friedrich B, Bryant FB (2009). The relationships among self‐esteem, stress, coping, eating behavior, and depressive mood in adolescents. Res Nurs Health.

[40] Hampl JS, Betts NM (1999). Cigarette use during adolescence: effects on nutritional status. Nutr Rev.

[41] Hanson MD, Chen E (2007). Socioeconomic status and health behaviors in adolescence: a review of the literature. J Behav Med.

[42] Dallongeville J, Marécaux N, Fruchart J-C, Amouyel P (1998). Cigarette smoking is associated with unhealthy patterns of nutrient intake: a meta-analysis. J Nutr.

[43] Fryer S, Waller G, Kroese BS (1997). Stress, coping, and disturbed eating attitudes in teenage girls. International Journal of Eating Disorders.

[44] Chen Z (2009). The health status of the left-behind children in rural China. Chinese Journal of Population Science.

[45] Guo L (2012). Migration and the well-being of left-behind children in China. *3518148.* United States.

[46] McKenzie D, Sasin M: Migration, remittances, poverty, and human capital: conceptual and empirical challenges. In: World Bank Policy Research Working Paper 4272. World bank; 2007

[47] Fund UNC’s (1998). The State of the World’s Children 1998.

[48] United Nations Children’s Fund (1990). Strategies for improving the nutrtional status of women and children in developing countries.

[49] Engle PL, Menon P, Haddad L (1999). Care and nutrition: concepts and measurement. World Dev.

[50] Miyazaki Y, Raudenbush SW (2000). Tests for linkage of multiple cohorts in an accelerated longitudinal design. Psychol Methods.

